# Comprehensive Transcriptome Sequencing Analysis of *Hirudinaria manillensis* in Different Growth Periods

**DOI:** 10.3389/fphys.2022.897458

**Published:** 2022-05-25

**Authors:** Huiquan Shan, Ke Ren, Jiasheng Liu, Saif ur Rehman, Xiuying Yan, Xiaocong Ma, Yalin Zheng, Tong Feng, Xiaobo Wang, Zhipeng Li, Weiguan Zhou, Chen Chuang, Mingkun Liang, Jinghui Zheng, Qingyou Liu

**Affiliations:** ^1^ Guangdong Provincial Key Laboratory of Animal Molecular Design and Precise Breeding, School of Life Science and Engineering, Foshan University, Foshan, China; ^2^ State Key Laboratory for Conservation and Utilization of Subtropical Agro- Bioresources, Guangxi University, Nanning, China; ^3^ Department of Cardiology, Ruikang Hospital Affiliated to Guangxi University of Chinese Medicine, Nanning, China; ^4^ THAI Natural Hirudin Co., Ltd., Bangkok, Thailand; ^5^ Guangxi Medical University Cancer Hospital, Nanning, China

**Keywords:** *Hirudinaria manillensis*, different growth periods, comprehensive transcription analysis, DEGs (differentially expressed genes), time series analysis

## Abstract

Medical leeches are widely been used in biochemical and clinical medical studies, helping to restore blood circulation to grafted or severely injured tissue. Mostly, adult leeches are being used in the traditional pharmacopeia, but the gene expression profiling of leeches in different growth periods is not well-reported. So, in this study, we used transcriptome analysis to analyze the comparative gene expression patterns of *Hirudinaria manillensis* (*H. manillensis*) in different growth periods, including larval, young, and adult stages. We constructed 24 cDNA libraries from *H. manillensis* larval, young, and adult stages, and about 54,639,118 sequences were generated, 18,106 mRNA transcripts of which 958 novel mRNAs and 491 lncRNAs were also assembled as well. Furthermore, the results of Gene Ontology (GO) and Kyoto Encyclopedia of Genes and Genomes (KEGG) enrichment analyses revealed that the differentially upregulated genes from the larval to adult stages were enriched in pathways such as cilium, myofibril, contractile fiber, cytoskeleton proteins, dilated cardiomyopathy, adrenergic signaling in cardiomyocytes, etc. Moreover, in the adult stages, a significant increase in the expression of the Hirudin-HM (*HIRM2*) genes was detected. In addition, our comparative transcriptome profiling data from different growth stages of *H. manillensis* also identified a large number of DEGs and DElncRNAs which were tentatively found to be associated with the growth of *H. manillensis*; as it grew, the muscle-related gene expression increased, while the lipid metabolism and need for stimulation and nutrition-related genes decreased. Similarly, the higher expression of *HIRM2* might attribute to the high expression of protein disulfide isomerase gene family (PDI) family genes in adulthood, which provides an important clue that why adult leeches rather than young leeches are widely used in clinical therapeutics and traditional Chinese medicine.

## Introduction

The medicinal leeches (*Annelida*, *Clitellata*, and *Hirudiniformes*) are categorized as aquatic predators that have widely been distributed across the globe and exhibited a variety of behavioral and physiological characteristics which are of great interest to evolutionary, biochemical, and pharmaceutical studies (Phillips et al., 2020). Based on their feeding habits, leeches are grouped into two major types, that is, non–blood-sucking and blood-sucking leeches (Chopin et al., 1998). *Hirudinaria manillensis* (*H. manillensis*) is an Asian buffalo leech, usually known as the Asian medicinal leech. *H. manillensis* is the most common blood-sucking leeches distributed in natural water of the South East Asian region and is broadly used in the Hirudo therapy (a therapy that bloodletting in certain tissues using live leeches to treat diverse ailments) (Elliott and Kutschera, 2011; Gileva and Mumcuoglu, 2013; Su et al., 2021). *H. manillensis* is one of the mainstream varieties used in traditional Chinese medicine in China (Liu et al., 2016; Zhang et al., 2013).

Leeches have an extended history of medicinal use and have been documented in ancient Greece, Rome, Arabia, and China, not only for bloodletting effects but also for inflammation, skin diseases, rheumatic pain, etc. (Hyson, 2005; Babenko et al., 2020; Şenel et al., 2020). The blood-sucking behavior of the medicinal leech allows it to remove the venous stasis and hematomas after reconstructive plastic surgery (Conforti et al., 2002; Riede et al., 2010). The active chemical secretions of *H. manillensis* into the host blood not only facilitate the blood-sucking behavior but also have important implications in medicine and clinical practice. Classically, hirudin-HM not only specifically binds to thrombin and causes anticoagulation, which enhances the fibrinolysis, but also has the functional role of anti–myocardial infarction, treatment of organic-type coronary arterial disease, and anti–deep vein thrombosis (De Filippis et al., 1995; Junren et al., 2021; Zhang et al., 2020). Furthermore, several other active substances with anticoagulant, anti-inflammatory, and analgesic properties have already been identified (Guan et al., 2019; Liu et al., 2019).

The advancement in genome sequencing enables researchers to explore at a molecular level. The development of sequencing technology results in the high-quality leech genomes continue to be published, and the study of leeches is becoming more comprehensive. The analysis of *H. manillensis* coding sequence (CDS) codon usage and related indices revealed that natural selection is the driving force behind the genetic evolution of *H. manillensis* (Guan et al., 2018). Adult *H. manillensis* is widely used in biochemical and clinical medical studies, but only a few studies have been reported on *H. manillensis* at different growth stages. So in the current study, we conduct transcriptome sequencing of *H. manillensis* at different growth periods (larval, young, and adult stages) to dissect the gene expression profiling during different growth stages, which would ultimately benefit to find the functional genes.

## Material and Methods

### *H. manillensis* Tissue Sampling

*H. manillensis* larval (10 days after birth), young (3 months after birth), and adult worms (2 years after birth) were collected from Qinzhou Zhanhong Chinese Medicinal Materials company. The leeches were immediately executed using scissors, and 24 samples were collected from the anterior and posterior segments of larval and young worms, while the adult samples were collected from oral suckers, gonads, caudal suckers, and the remaining worm body. Moreover, each time, five *H. manillensis* were collected and preserved in water bottles filled with pure water for subsequent histological examination.

### *H. manillensis* Histomorphology

The histomorphological characteristics of *H. manillensis* were observed by conventional paraffin embedding, sectioning, and hematoxylin and eosin staining. A microscope (EVOS FL Auto) was used to examine leech gonads and muscle histomorphology, and images were captured using an EVOS Auto microphotographic system.

### Constructing cDNA Library and Sequence Data Analysis

Total RNA was extracted from different parts of leeches at different developmental stages using TRIzol reagent (Invitrogen Corp., Carlsbad, CA, United States), and RNA purifications were performed using the RNeasy Mini Kit (Qiagen, Chatsworth, CA, United States). The sequencing libraries were constructed using the NEBNext Ultra RNA Library Prep Kit for Illumina (NEB, United States) following the manufacturer’s recommendations, and the libraries were sequenced on an Illumina HiSeq 4000 platform to generate 150 bp paired-end reads. Each sample quality was controlled with Fastp (v0.20.1) (Chen et al., 2018). Furthermore, we used HISAT2 (v2.2.1) to map the reads from each sample to the reference genome, and Samtools was used to sort and convert the SAM files to BAM; we use the “merge” parameter of the Samtools tool to combine the data from different parts together (Kim et al., 2015). Moreover, StringTie (v2.1.4) was utilized to assemble and merge the transcripts of each sample (Pertea et al., 2015), and those merged transcripts were then subjected to Gffcompare (v0.11.2) to compare with the reference annotation file in GTF, and transcript abundances were estimated using StringTie with options “-e -B -p 20” (Pertea and Pertea, 2020). The results of abundances were stored ended with “balltown,” and prepDE.py was used to compare the folders.

### Analysis of Differentially Expressed Genes

The gene expression was predicted in fragments per kilobase of transcript per million fragments mapped (FPKM), and differentially expressed genes (DEGs) among three stages were detected using the DESeq2 (v1.32.1) package (Love et al., 2014). Genes with FDR (false discovery rate) values < 0.05 and |log^2^FC| ≥ 1 were considered as DEGs between two stages. Furthermore, the simultaneous mapping of differentially expressed genes was carried out using the ggplot2 (v3.3.5) package (Wickham, 2016).

### Sample Time-Series Analysis

The time course sequencing data analysis (Tcseq) (v1.18.0) software was used to analyze the DEGs at three time points and clusters and graphically illustrated the different gene expression patterns at different developmental stages (Wu and Gu, 2021). The genes with FDR < 0.05 and |log^2^FC| ≥ 1 were considered DEGs between the two stages.

### The Identification and Analysis of lncRNAs

The following highly stringent criterion was used to screen potential lncRNAs: 1) transcripts with >200 nt length were selected; 2) the transcripts were annotated as “i”, “u”, and “x” according to the cuffcompare classes; 3) the coding potential calculator (CPC2) score was “noncoding”; 4) transcripts with an open-reading frame (ORF) of more than 50 amino acids were removed; and 5) transcripts with known protein-coding domains were excised by aligning to the Swiss-Prot database. Furthermore, as a *cis*-regulator, lncRNA has the potential to influence the expression of adjacent genes; thus, a coexpression network of the candidate lncRNAs and their upstream or downstream 10 kb mRNAs was constructed. The connectivity and enrichment analysis was performed with the Position Frequency Matrix (Azlan et al., 2019).

### Functional Enrichment Analysis of Differentially Expressed Genes

Gene Ontology (GO, http://www.geneontology.org/) and KEGG (Kyoto Encyclopedia of Genes and Genomes) analyses of the differentially expressed were performed using clusterProfiler (statistical analysis and visualization of functional profiles for genes and gene clusters). The value of FDR < 0.05 was considered significantly enriched.

### Validation by Real-Time Quantitative PCR

The redundant samples from transcriptome sequencing were utilized for qRT-PCR analysis. The first-strand cDNA was synthesized with 1 μg total RNA using a HiScript^®^ III-RT SuperMix for qPCR (+gDNA wiper) kit (Vazyme, R323-01). Primers of the chosen genes were designed by using oligo 7 software (Supplementary Table S3) (Rychlik, 2007). The expression levels of selected genes were quantified using Universal SYBR qPCR Master Mix (Vazyme, Q711-03), and data were acquired using an RT-PCR instrument (ABI 7500), according to the manufacturer’s instructions. For each detection cycle, three biological and three technical replicates were used. The relative gene expression was calculated using the 2^−△△Ct^ method using RO21 as the reference gene to normalize the relative mRNA expression levels (Schmittgen and Livak, 2008).

### Statistical Analysis

All the obtained data were statistically analyzed by using Student’s t-test and analysis of variance (ANOVA) with DUNCAN’s Multiple Range Test (DMR) in SPSS 17.0 software (IBM Corp., Armonk, NY, United States) (SPSSAU, 2017). The *p* values < 0.05 were considered statistically significant.

## Results

### *H. manillensis* Body Weight and Transverse Paraffin Section Comparison at Different Growth Periods

**FIGURE 1:**
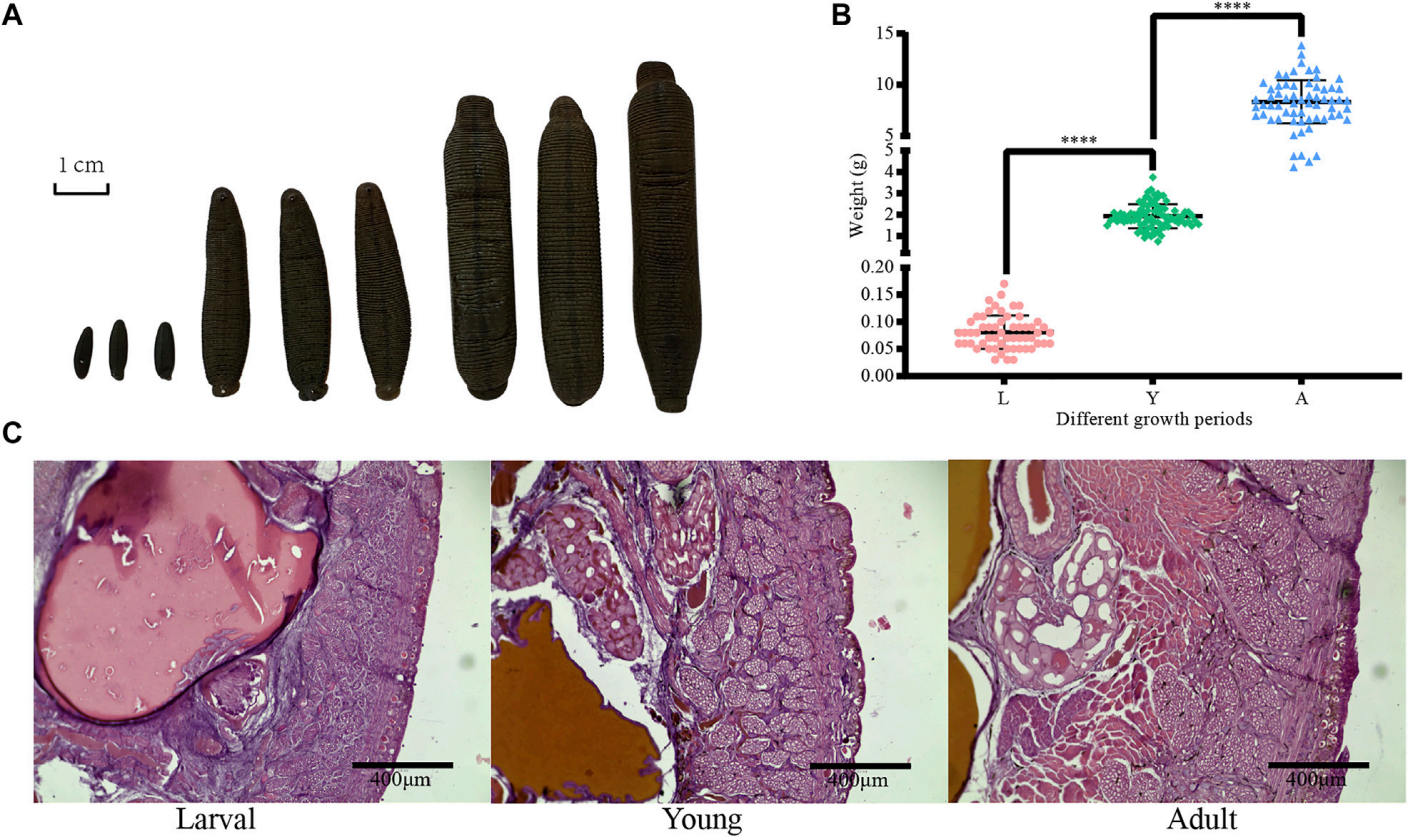
Phenotypic data of *H. manillensis*. **(A)** Appearance of the different growth periods of *H. manillensis*. **(B)** Body weights of different growth periods of *H. manillensis*. **(C)** Transverse paraffin sections of the skin layers of *H. manillensis* at different growth periods.

First, the collected *H. manillensis* worms at larval (L), young (Y), and adult (A) stages were fixed with formaldehyde and then projected as shown in Figure 1A. As presented in Figure 1B, a highly significant difference (*p < 0.0001*) was observed in the bodyweight of *H. manillensis* at different growth stages, with the larval stage weighing 0.081 ± 0.031 g, young 1.922 ± 0.563 g, and adults 8.329 ± 2.100 g. Furthermore, we also compared the transverse paraffin sections (Figure 1C) of *H. manillensis* (larval, young, and adult), and only the adult *H. manillensis* had a significant muscle bundle tissue, while it was not spotted in the larval and young stages.

### RNA-Sequencing Profile of Larva, Young, and Adult *H. manillensis*

We have constructed a total of 24 cDNA libraries for larva, young, and adult *H. manillensis* worms (HMLB1, HMLB2, HMLB3, HMLF1, HMLF2, HMLF3, HMYB1, HMYB2, HMYB3, HMYF1, HMYF2, HMYF3, HMO1, HMO2, HMO3, HMG1, HMG2, HMG3, HMP1, HMP2, HMP3, HMB1, HMB2, and HMB3) (Supplementary Table S1). A total of 54,639,118 sequences were obtained, with a GC content ranging between 43% and 45% (Supplementary Table S2). Furthermore, the sequenced raw data from different growth periods were combined to obtain three samples for each stage (L1, L2, and L3; Y1, Y2, and Y3; and A1, A2, and A3).

**FIGURE 2:**
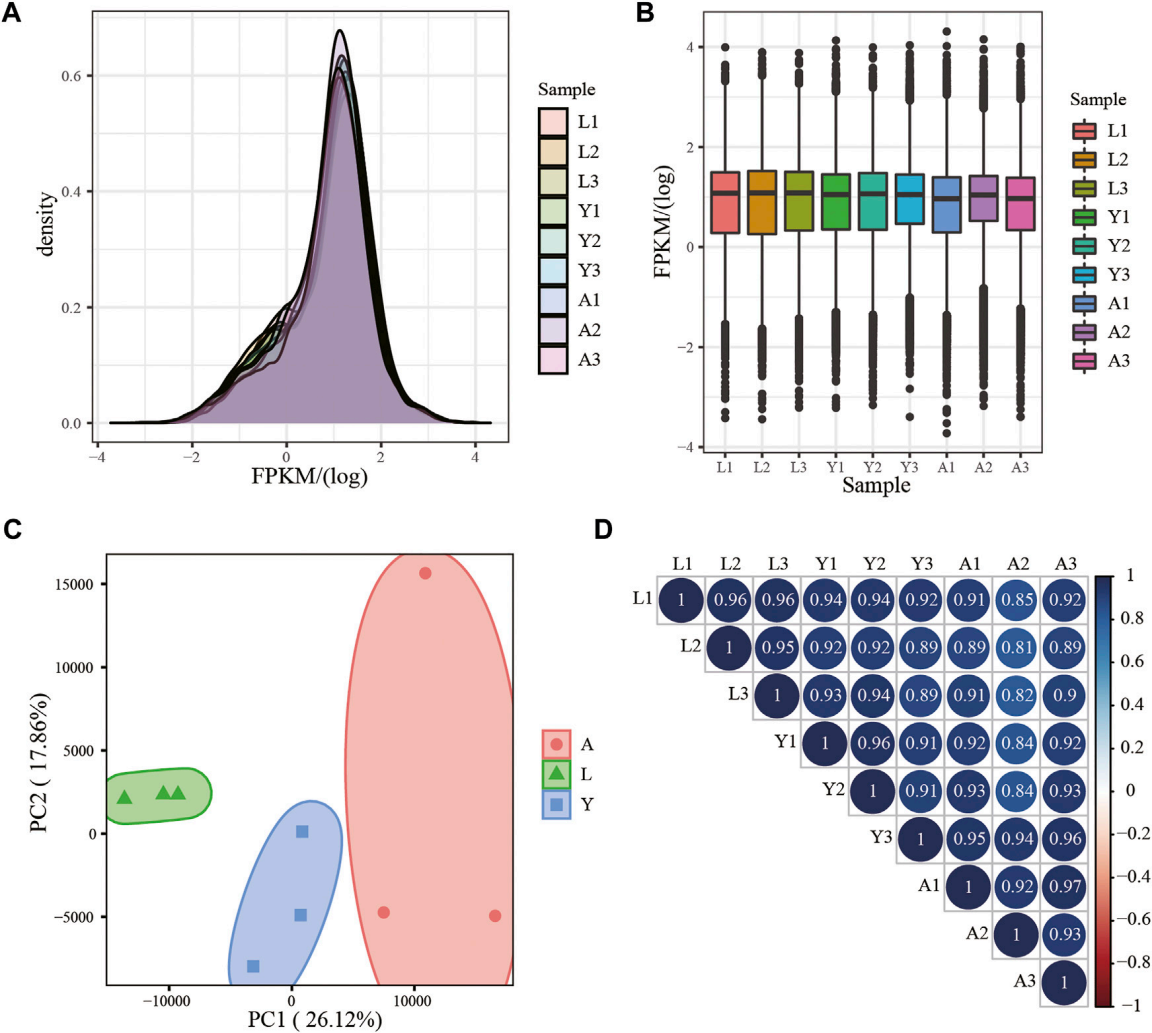
mRNA expression analysis. **(A)** Density distribution of mRNAs was according to log10 (FPKM); **(B)** Nine sample expression (L1, L2, L3, Y1, Y2, Y3, A1, A2, and A3) violin plot, which was replaced by log10 (FPKM). **(C)** PCA distribution of nine samples; **(D)** Sample relationship cluster analysis.

Using RNA-seq, about 18,106 mRNAs transcripts were detected, and their expression is presented in FPKM values and the FPKM distribution of mRNAs is shown in Figure 2A, whereas the expression of different samples is presented in the Box chart (Figure 2B). All the nine samples were divided into three parts by PCA analysis, which demonstrated satisfactory repeatability (Figure 2C) and then, a relationship cluster diagram was constructed to represent the relationship between samples (Figure 2D) intuitively. Sequences exhibited a reliable clustering effect, ensuring the veracity of the subsequent analysis.

### Analysis of Differentially Expressed Genes

**FIGURE 3:**
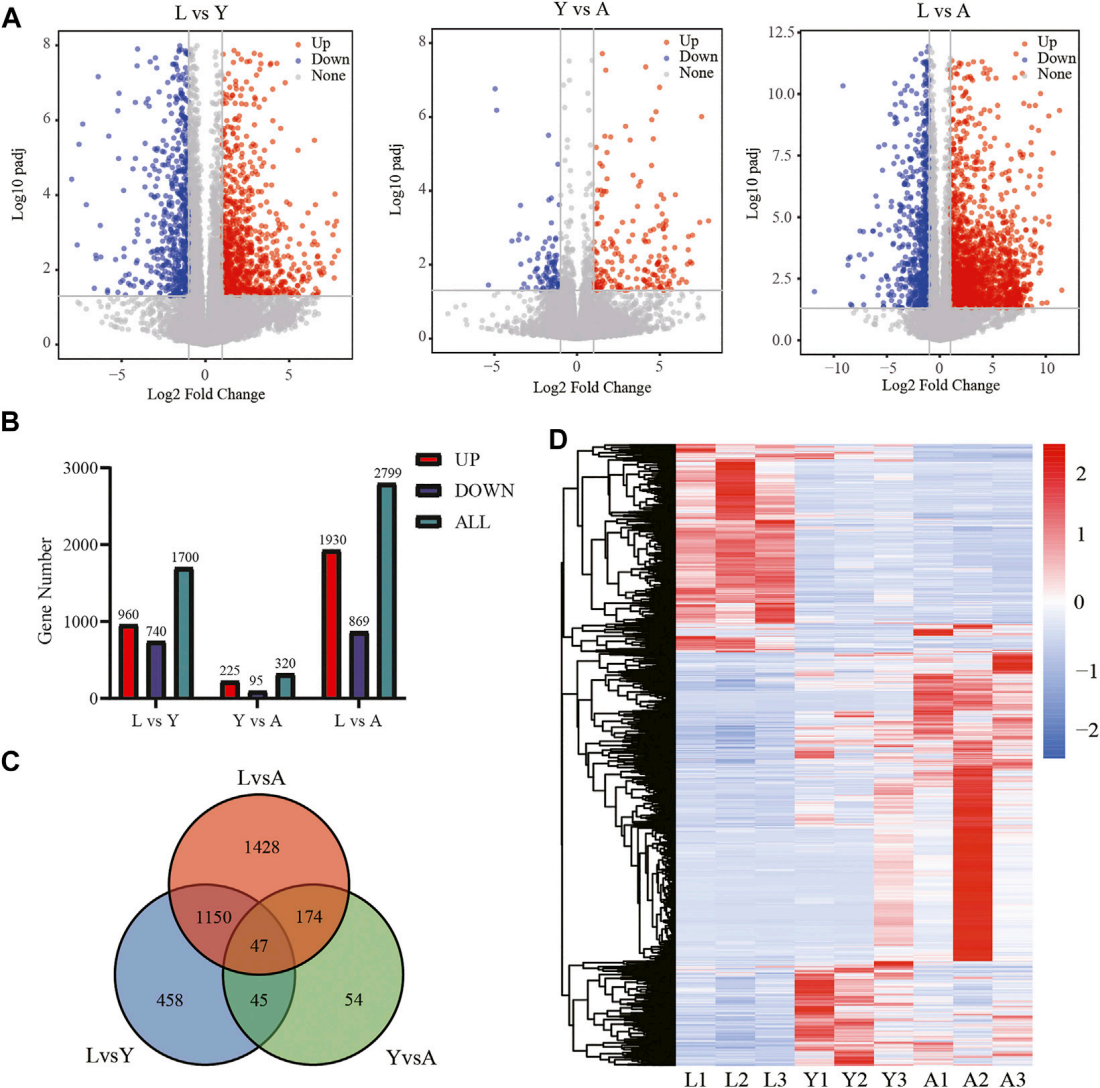
Differential expression analyses of mRNAs. **(A)** Differential genetic volcano map Comparison of two different growth periods; **(B)** Differential gene statistics of different growth periods; **(C)** Venn plot of DEGs; **(D)** Differential gene cluster analysis.

We used an FDR value < 0.05 and |log^2^FC| ≥ 1 as the criteria to screen the DEGs by comparing pairwise differences between larva, young, and adult *H. manillensis*. We obtained 1,700 DEGs after comparing the L *vs*. Y, 320 from Y *vs*. A, and 2,799 in L *vs*. A (Figures 3A,B). We presented VENN plots of these DEGs from L, Y, and A growth stages (Figure 3C), and a total of 47 intersections of DEGs were assessed with 3,356 DEGs, and their expression patterns were illustrated in a heat map using clustering analysis (Figure 3D). We found that both L *vs*. Y and L *vs*. A had high differentially expressed genes during the growth of *H. manillensis*, while some of the differentially expressed genes were significantly reduced during Y *vs*. A. Therefore, the growth and development of *H. manillensis* gradually stabilized when they reached the young stage.

### Sample Time-Series Analysis of the Differentially Expressed Genes

**FIGURE 4:**
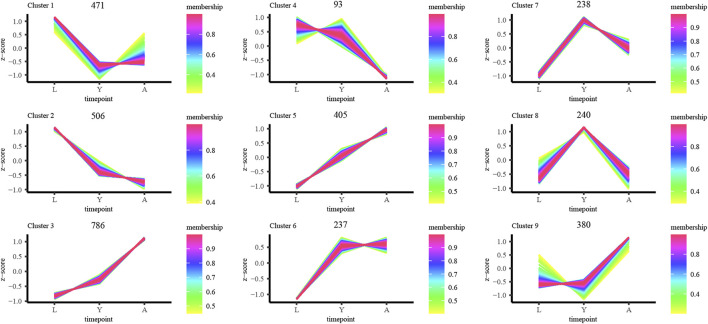
Sample time-series analysis of DEGs. The horizontal axis represents different growth periods, and the vertical axis represents normalized gene expression.

Furthermore, we examined the DEG expression trends and screened the DEGs (gene expression profile indicated by FPKM) using FDR < 0.05, and |log^2^FC| ≥ 1 to show the development of *H. manillensis* at various growth phases. We restricted our study to nine clusters of which five had overall upregulated trends and four downregulated trends, and the majority of the genes were enriched in the overall upregulated trend, especially the Cluster 3 which enriched a total of 786 DEGs (Figure 4). These findings can effectively reveal the gene expression patterns of *H. manillensis* in different growth phases.

### Gene Ontology and Kyoto Encyclopedia of Genes and Genomes Pathway Enrichment Analyses of the Differentially Expressed Genes

**FIGURE 5:**
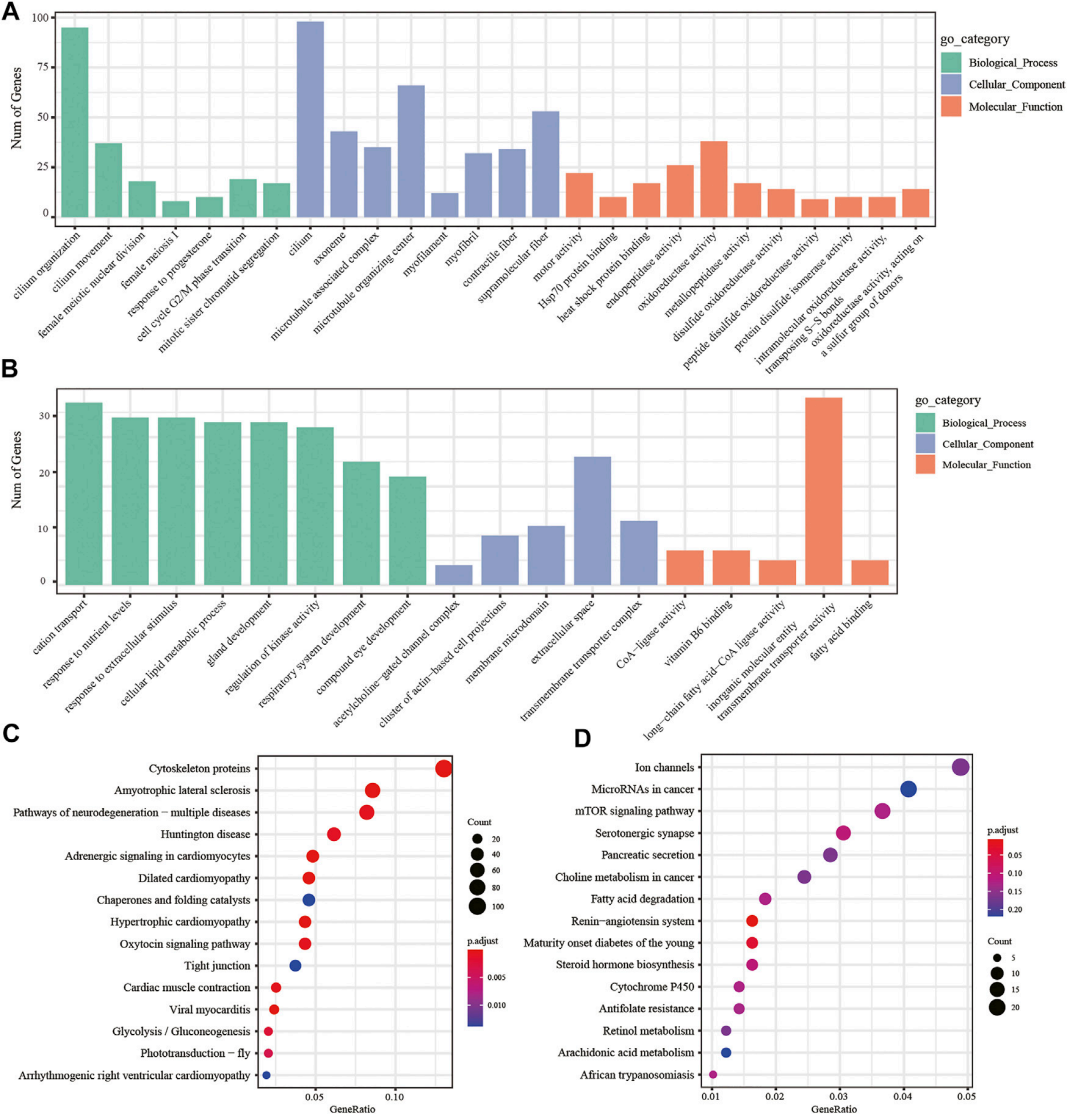
DEG enrichment analysis in the LvsA period. GO enrichment analysis of **(A)** upregulated and **(B)** downregulated differential genes during the LvsA period; KEGG enrichment analysis of **(C)** upregulated and **(D)** downregulated differential expressed genes during LvsA period.

In addition, we performed two-by-two comparisons between larval, young, and adult stages of *H. manillensis* at FDR < 0.05 and |log^2^FC| ≥ 1, and the genes having the value of log^2^FC ≥ 1 were considered upregulated genes and downregulated with log^2^FC ≤ −1 and then performed the GO and KEGG enrichment analyses. Using the GO enrichment analysis, we obtained significant enrichment of differentially upregulated and downregulated genes in three components: biological processes, cellular components, and molecular functions of the larval and adult stages (Figures 5A,B). These upregulated and downregulated genes were found to be enriched in biological processes such as cilium organization, cilium movement, cell cycle G2/M phase transition; in cellular components such as cilium, myofilament, microtubule-associated complex; and molecular functions such as motor activity, oxidoreductase activity, and disulfide oxidoreductase activity. Moreover, the KEGG enrichment analysis of the differentially upregulated and downregulated genes for larval and adult stages is shown in Figures 5C,D, where upregulated genes were mainly enriched in cytoskeleton proteins, dilated cardiomyopathy, adrenergic signaling in cardiomyocytes, and glycolysis/gluconeogenesis, while downregulated genes were mainly enriched in the renin–angiotensin system, maturity onset diabetes of the young, mTOR signaling pathway, fatty acid degradation, cytochrome P450, and other pathways. Also, the GO and KEGG profiles of *H. manillensis* L vs. Y and Y vs. A differential expressed genes are shown in Supplementary Figures S1, S2.

### The Growth and Development Related Gene Family’s Expression Pattern Analysis

**FIGURE 6:**
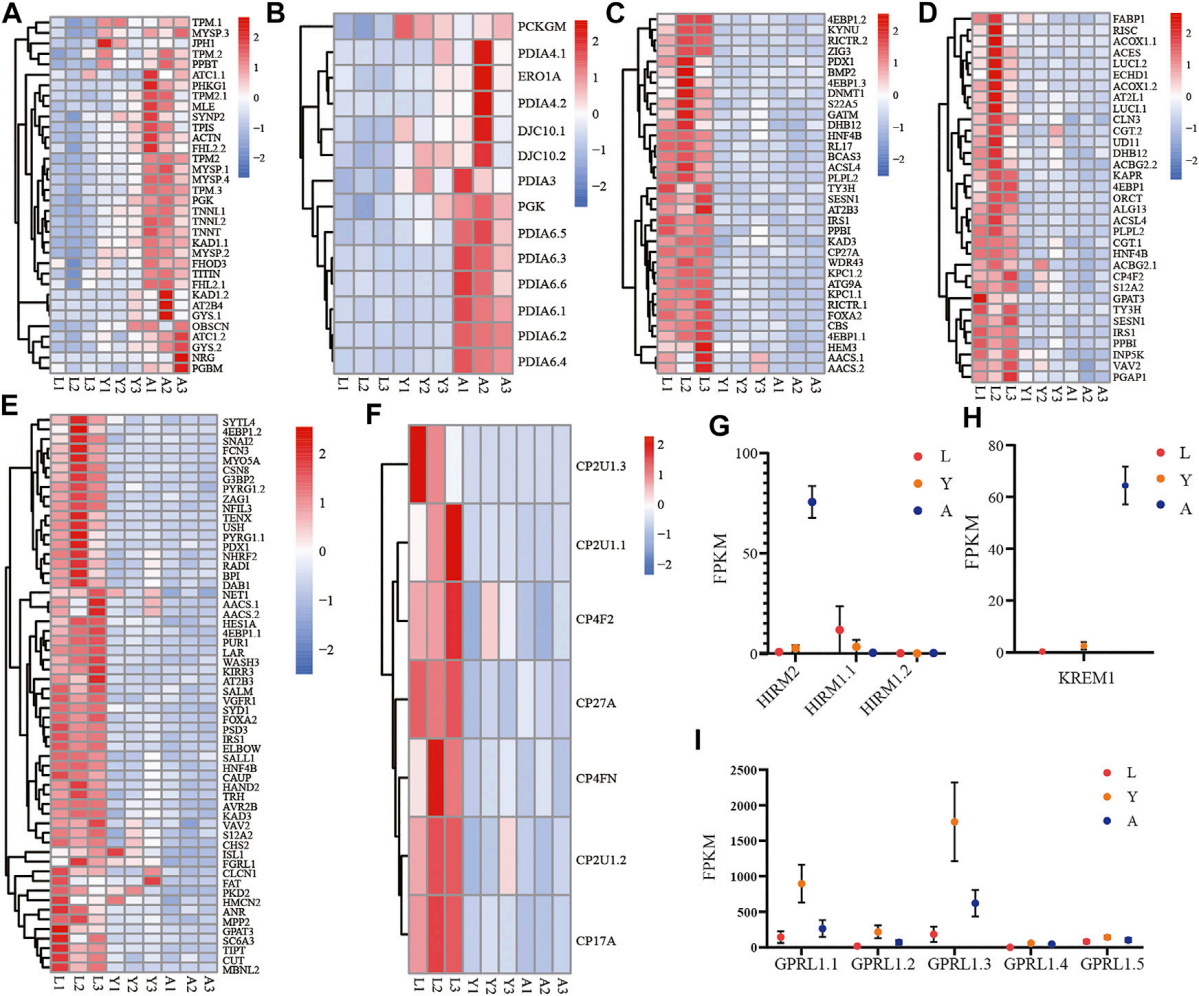
Expression of important gene families related to growth and development. The gene family expression of **(A)** muscle cellular component–related genes; **(B)** disulfide oxidoreductase activity; **(C)** response to stimulation and nutrition-related genes; **(D)** cellular lipid metabolism–related genes; **(E)** gland development, respiratory system development, and compound eye development-related genes; **(F)** cytochrome P450–related genes; **(G)** *HIRM2* (hirudin-HM) and *HIRM1* (hirudin); **(H)** *KREM1*; **(I)** *GPRL1* in three different growth periods of *H. manillensis.*

Likewise, among the biological components examined by GO enrichment, the expression of DEGs in the myofibrillar and contractile fiber pathways exhibited a progressive upregulation during the L-Y-A period (Figure 6A). The gene family expression related to the disulfide oxidoreductase activity pathway in molecular functions tends to be gradually upregulated (Figure 6B), and the genes of the PAI gene family were the majorly detected genes. There is also a progressive downregulation of genes that are enriched in response to nutrient levels, extracellular stimuli, and in cellular lipid metabolism (Figures 6C, D). The downregulated genes were also linked with development-related pathways such as gland development, respiratory system development, and compound eye development (Figure 6E). The KEGG enrichment analysis findings at the L-A stage revealed that the significantly downregulated genes were associated with the cytochrome P450 family pathway, which tended to be progressively lower in the *H. manillensis’s* three growth stages (Figure 6F), notably for the *CP2U1* gene. Furthermore, we observed a significantly higher expression of the *HIRM2* and *KREM1* genes throughout the adult stage and an overall lower expression of the *HIRM1* gene family across the three developmental periods (Figures 6G,H). The *GPRL1* gene family, which is associated with spermatogenesis, was also found to be highly expressed during the L-Y period, while decreased expression was seen during the Y-A period (Figure 6I).

### Analysis of Differential Expressed lncRNAs in *H. manillensis* During Different Growth Periods

**FIGURE 7:**
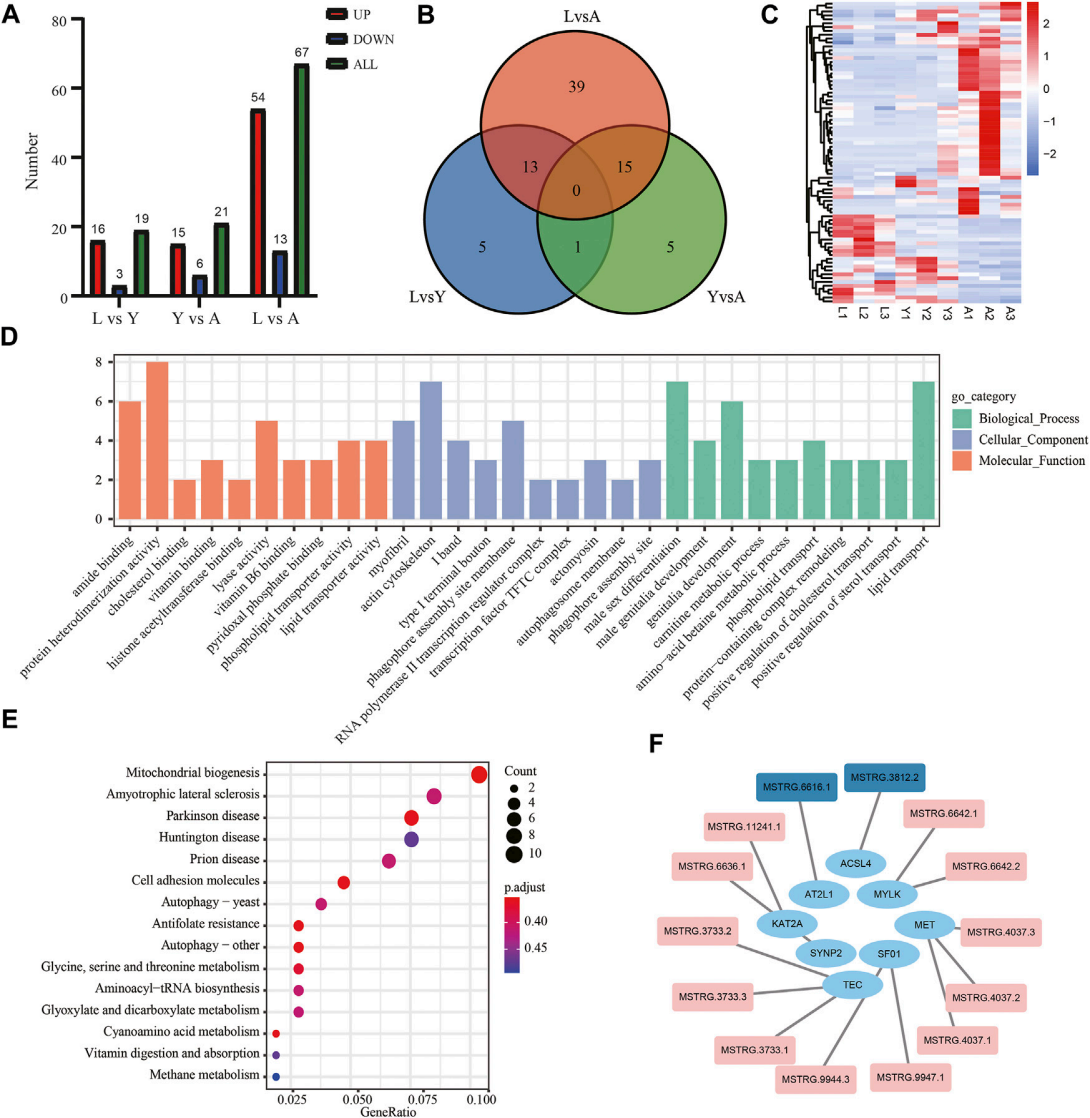
Screening and enrichment analysis of DElncRNAs in *H. manillensis* during different growth periods. **(A)** DElncRNAs statistics of different growth periods; **(B)** Venn plot of DElncRNAs; **(C)** DElncRNA cluster analysis; **(D)** GO enrichment graph shows the GO analysis of the target gene of DElncRNAs predicted by *cis*; **(E)** KEGG analysis was used to uncover the role of DElncRNA target gene predicted by *cis*; **(F)** lncRNA target genes predicted by *cis* (red indicates DElncRNAs).

The DElncRNAs were screened by comparing pairwise differences from three different growth periods of *H. manillensis* using FDR < 0.05 and |log^2^FC| ≥ 1 as a criterion, and a total of 107 DElncRNAs were detected of 19 DElncRNAs from L vs. Y, 21 from Y vs. A and 67 from L vs. A (Figure 7A). VENN plot presentation of DElncRNAs for these different growth periods is shown in Figure 7B. We obtained a total of 78 DElncRNAs and were shown in a heat map by clustering analysis (Figure 7C). Moreover, GO enrichment analysis showed that these DElncRNA target genes were mainly enriched in genitalia development, actin cytoskeleton, and actomyosin (Figure 7D) and might target mRNA to affect mitochondrial biogenesis, cyanoamino acid metabolism, and Parkinson’s disease (Figure 7E). The 491 lncRNAs were predicted by *cis* prediction, and there were 14 lncRAs that could target the eight mRNAs (Figure 7F).

### Confirmation of Gene Expression With Quantitative PCR

**FIGURE 8:**
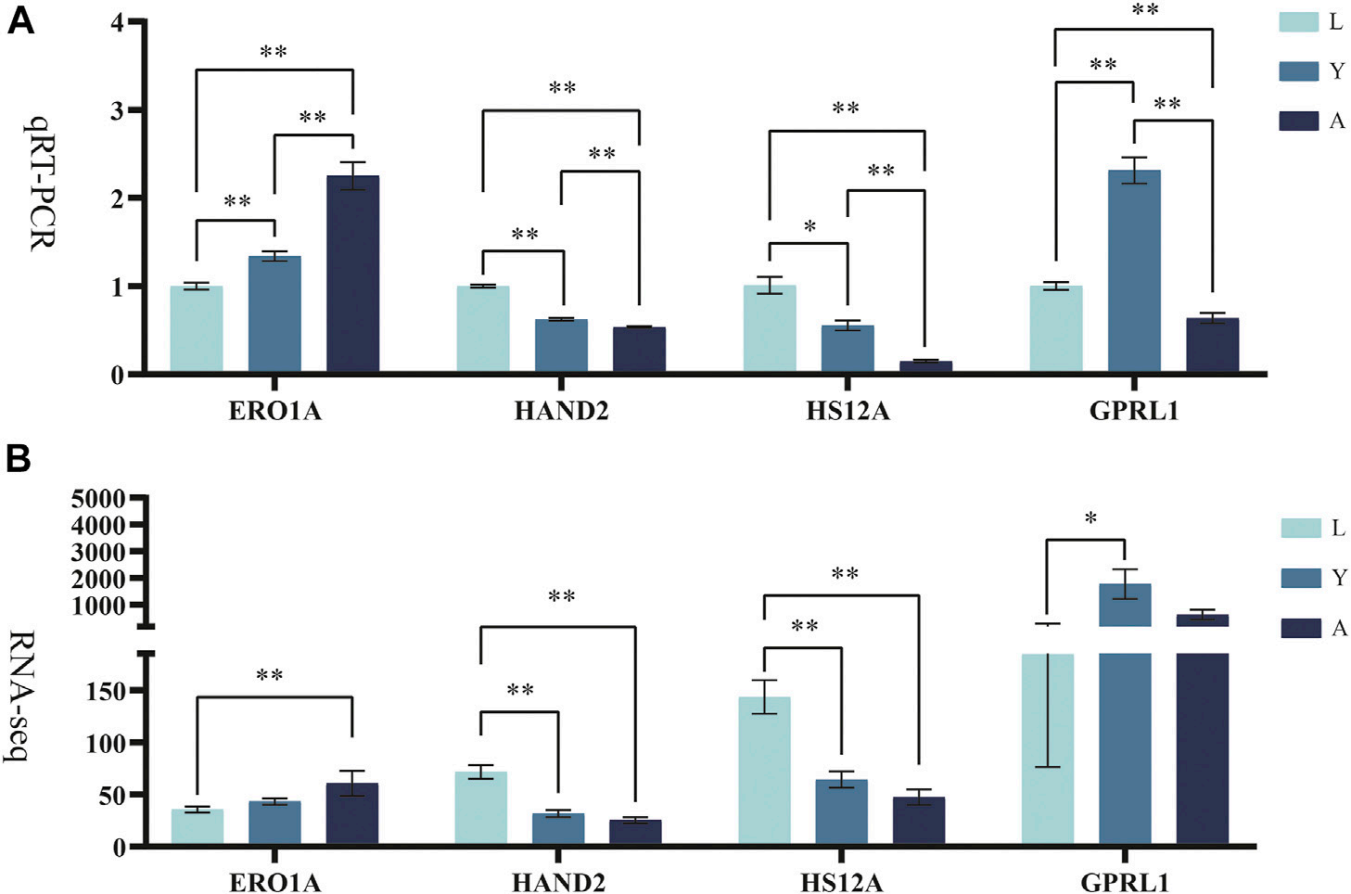
Validation of candidate genes. **(A)** qRT-PCR, *RO21* was used as the reference gene. **(B)** FPKM represents RNA-seq relative expression.* means *p* < 0.05, ** means *p* < 0.01.

The differentially expressed genes (*ERO1A*, *HAND2*, *HS12A*, and *GPRL1*) were selected for Q-PCR quantification in order to confirm their differential expression in different growth periods of *H. manillensis* obtained by RNA-seq. The expression of these genes was confirmed by Q-PCR (Figures 8A,B).

## Discussion

*H. manillensis* is an important constituent of traditional medicines and has long been used in the medical field to treat a number of ailments; thus, it is imperative to disclose the genetics of the *H. manillensis* throughout its dynamic process of development. So, we selected three growth stages (L, Y, and A) of *H. manillensis* and performed the transcriptome sequencing to better understand the developmental changes that occur during *H. manillensis* growth. At the three time nodes, we discovered a total of 3,356 DEGs and 78 DElncRNAs. About 2,799 and 320 differentially expressed genes have been detected in L-A and Y-A growth phases, respectively, indicating that by the time *H. manillensis* reached the young insect stage, its structural organization and body functions had matured.

One of the most fascinating aspects of *H. manillensis* research is its unique secretion of *HIRM2* (hirudin-HM), which is structurally identical to that of natural hirudin-HM (*HIRM2*), a disulfide-bonded cross-linked polypeptide of 8 kDa size that could bind specifically to thrombin to produce anticoagulant effects (De Filippis et al., 1995; Scacheri et al., 1993; Vindigni et al., 1994). In contrast, this disulfide bond formation is also one of the most important posttranslational modifications in hirudin synthesis which not only ensure the biological activity of the protein but is also helpful in categorizing the protein into a specific class or family (Kaas and Craik, 2015). In our study, the upregulated genes were enriched in the disulfide oxidoreductase activity pathway, and these genes were members of the *PDI* gene family. Although *PDI* can catalyze the formation, isomerization, or reduction of disulfide bonds in nascent peptide chains, it can only transmit the redox equivalents, while *ERO1A*, a flavoprotein with a FAD cofactor, can utilize oxygen molecules as a substrate and provides the oxidative equivalents necessary for the production of disulfide bonds in nascent peptide chains in the endoplasmic reticulum lumen (Tu and Weissman, 2002). In this study, we found that *HIRM2* was the only highly expressed gene in adult *H. manillensis*, which might be associated with the higher expression of genes such as *PDI* and *ERO1A* in adulthood, which assist in the disulfide bond synthesis of *HIRM2*. This result also provides the theoretical basis for the medicinal use of adult *H. manillensis*.

Muscular growth is essential for an organism’s structural organization, which aids in maintaining its life activities. Furthermore, we have found significantly more muscles in adult *H. manillensis* than in the larvae and young. The upregulated genes of *H. manillensis* L vs. A and Y vs. A were enriched in myofibril and contractile fiber in the cellular components. Among those, the *MYSP* paramyosin is a fundamental structural component of molluscan muscle thick filaments and plays an important role in the “capture” mechanism, which allows invertebrates to have continuous muscle contraction (Vyatchin et al., 2019; Watabe and Hartshome, 1990). The *TPM* (tropomyosin) also contributes to proper functioning of the actin cytoskeleton, and its role is important for the survival of a wide range of organisms (Gunning et al., 2008). The binding of *TNNT* calcium to troponin activates the myosin ATPase activity in molluscan scallop rhabdomyosus (Wei and Jin, 2011). The MSTRG.3812.2 targeted gene *SYNP2* which is part of cellular actin organizing center (Schroeter et al., 2013). Our study is also in line with previous studies because the muscle-related genes were highly expressed in adult *H. manillensis*.

Moreover, downregulated genes were prioritized in response to food levels and external stimuli in the biological process during both the L *vs*. Y and L *vs*. A stages of *H. manillensis* development. To further investigate this phenomenon, we mined for differential genes in the pathway. First, we found that the genes such as *ACSBG2*, *EIF4EBP1*, MSTRG.6616.1 targeted gene *AT2L1*, and MSTRG.3812.2 targeted gene *ACSL4* in the cellular lipid metabolism pathway were highly expressed in the larval period. Therefore, the *H. manillensis* could enhance the cellular lipid metabolic process during the larval stage due to the higher energy need for growth and development, which in turn increases the demand for nutrients (Guo et al., 2018; Singh et al., 2019a; Tsai et al., 2016; Yang et al., 2021a), and the nutritional requirements of the *H. manillensis* can be decreased with reduced lipid metabolism in cells. Leech sensory cells have characteristic similarities to vertebrate hair cells, and it has earlier been reported that overexpression of the *KREM1* gene negatively affects the number of mechanosensory cells formed in zebrafish neuromas, and the zebrafish lacking the Kremen1 protein can develop more hair cells per neuromast than wild-type fish (Meiser et al., 2019; Mulvaney et al., 2016; Pellegrino et al., 2005). Therefore, the high expression of *KREM1* in the adult stage of *H. manillensis* leads to a decrease in mechanosensory cells and reduces the response to stimuli. It is also interesting to note that the cytochrome P450 family is highly expressed in response to starvation stress in *H. manillensis* when it has a high demand for nutrients due to growth requirements. The most important one is the higher expression of the cp2u1 gene family, which (or family 2, subfamily U, polypeptide 1) encodes an enzyme that can catalyze the hydroxylation of arachidonic acid (AA) and other long-chain fatty acids to prevent oxidative stress by activating the peroxisome receptor *γ* (Wang et al., 2006). In light-stressed unfed corals, the *CP2U1* gene has been found to lead to a cascade of metabolic issues downstream of oxidative stress (Levy et al., 2016). In the KEGG pathway, these genes are also enriched in cytochrome P450. Similarly, many other crucial genes were also expressed during the growth of *H. manillensis*, which have their interaction with the development process, such as *ZAG1* involved in neuronal differentiation and pharyngeal development, and the *ELB2* gene involved in organ development (Dorfman et al., 2002; Wacker et al., 2003). Particularly, *GPRL1* expression was higher at first and then gradually reduced in *H. manillensis* growth, and previous studies have indicated that *GPRL1* is mainly expressed in the testis or involved in the fertilization process and has an important role in the mating and reproduction process (Gibbs et al., 2010), while the decreased expression in adult *H. manillensis* may be due to the fact that *H. manillensis* is a hermaphrodite, which decreases sperm production during the adult egg-laying period.

DElncRNAs are functionally enriched mainly in the actin backbone and reproductive organ development. In the actin backbone, *SYNP2*, as a target gene of MSTRG.6636.1, is the component of the cellular actin organizing center (Schroeter et al., 2013). The *KAT2A* as a target gene of MSTRG.11241.1 is involved in heart and limb development and craniofacial cartilage and bone growth and differentiation in zebrafish and mice (Ghosh et al., 2018; Sen et al., 2018). The *MYLK* as a common target gene of MSTRG.6642.1 and MSTRG.6642.2 is a binding protein for actin, myosin, and CaM (Khapchaev and Shirinsky, 2016). During the reproductive organ development, *MET* plays an important role in testis cord formation as a target gene of MSTRG.4037.1, MSTRG.4037.2 and MSTRG.4037.3 (Ricci et al., 1999). *SF01* is a target gene of MSTRG.9947.1 and MSTRG.9944.3 and the targeted disruption of *SF01* in mice prevent gonadal and adrenal development and leads to male to female sex reversal (Ozisik et al., 2003). The target genes *TEC* of MSTRG.3733.1, MSTRG.3733.2, and MSTRG.3733.3 are invaluable for head degeneration during embryogenesis and ring tube development during oogenesis (Roulier et al., 1998). In addition, among some non-differential lncRNAs, *AT2L1* as the MSTRG.6616.1 target gene and *ACSL4* as the MSTRG.3812.2 target gene were found to be involved in lipid metabolism and phospholipid metabolism in *H. manillensis* (Singh et al., 2019b; Yang et al., 2021b).

## Conclusion

Through transcriptome analysis, the current study comprehensively explored the development of the *H. manillensis* at different growth stages, and 3,256 DEGs with 78 DElncRNAs were identified, and several DEGs were enriched in upregulation trends detected by time-series analysis. At different growth stages, we found that as the *H. manillensis* grew, the muscle-related gene expression increased, while a decreased expression was observed for lipid metabolism and the need for stimulation and nutrition. We found that DElncRNAs are functionally enriched mainly in the actin backbone and reproductive organ development. Specifically, we also found a higher expression of the hirudin-HM in adulthood. Taken together, these results could provide a theoretical basis for the breeding and subsequent medicinal use of *H. manillensis* in the future.

## Data Availability

The datasets presented in this study can be found in online repositories. The names of the repository/repositories and accession number(s) can be found below: https://www.ncbi.nlm.nih.gov/bioproject/, PRJNA762643.

## Abbreviations

L, larval; Y, young; A, adult; DEGs, differentially expressed genes; DElncRNAs, differentially expressed lncRNAs.
