# Supplementary material for: Comprehensive Transcriptome Sequencing Analysis of Hirudinaria manillensis in Different Growth Periods
Source: Front Physiol. 2022 May 25;13:897458. doi: 10.3389/fphys.2022.897458 (PMC9174698; doi:10.3389/fphys.2022.897458)
Supplement: Supplementary file 1 [file DataSheet2.docx]

**Supplementary information**

Supplementary information accompanies this paper as follow:

Additional file 4: Figure S1. Differential gene enrichment in LvsY period

Additional file 5: Figure S2. Differential gene enrichment in YvsA period


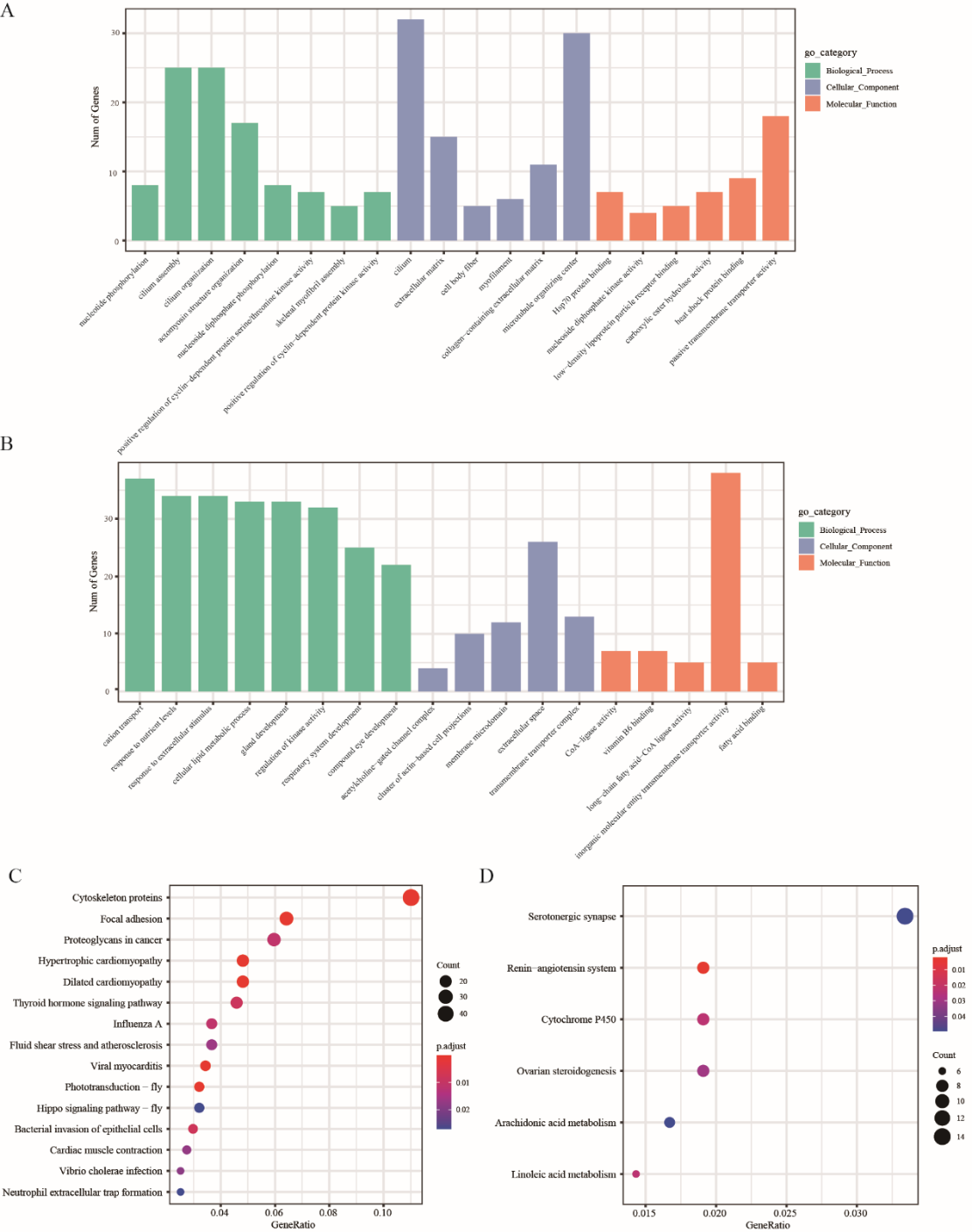


Figure S1. Differential gene enrichment analysis in LvsY period. (A) GO enrichment analysis of up-regulated differential genes during LY period; (B) GO enrichment analysis of down-regulated differential genes during LY period; (C) KEGG enrichment analysis of up-regulated differential genes during LY period; (D) KEGG enrichment analysis of down-regulated differential genes during LY period.


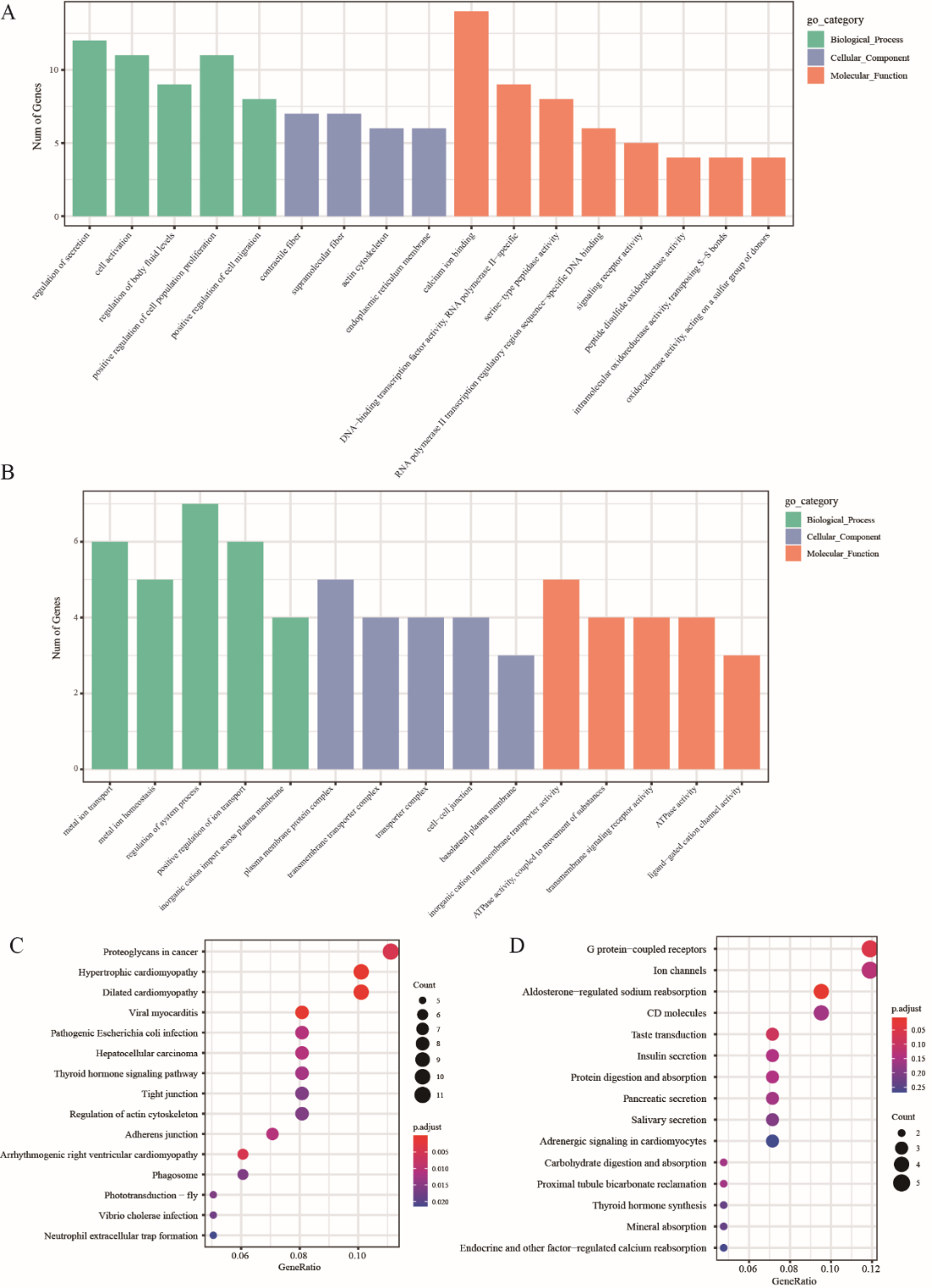


Figure S2. Differential gene enrichment analysis in YvsA period. (A) GO enrichment analysis of up-regulated differential genes during YA period; (B) GO enrichment analysis of down-regulated differential genes during YA period; (C) KEGG enrichment analysis of up-regulated differential genes during YA period; (D) KEGG enrichment analysis of down-regulated differential genes during YA period.
